# Transcriptome Reveals Growth Responses of *Populus qamdoensis* Under Blue and Green Films

**DOI:** 10.3390/biology14121658

**Published:** 2025-11-24

**Authors:** Xiaolin Zhang, Rong Xu, Cai Wang, Shihai Zhang, Lihong Zhao, Ning Zhao, Yulan Xu, Dan Zong

**Affiliations:** 1 Forest Resources Exploitation and Utilization Engineering Research Center for Grand Health of Yunnan Provincial Universities, Southwest Forestry University, Kunming 650224, China; sunnyxlz27@163.com (X.Z.); xxdr@swfu.edu.cn (R.X.); w413220@swfu.edu.cn (C.W.); zhangshihai@swfu.edu.cn (S.Z.); zhaolihong@swfu.edu.cn (L.Z.); lijiangzhn@163.com (N.Z.); xuyulan@swfu.edu.cn (Y.X.); 2 College of Forestry, Southwest Forestry University, Kunming 650224, China; 3 College of Biological Science and Food Engineering, Southwest Forestry University, Kunming 650224, China

**Keywords:** *Populus qamdoensis*, light quality, growth and development, metabolism pathways, photoreceptor

## Abstract

*Populus qamdoensis* cuttings were treated with transparent colorless film (WF), blue film (BF), and green film (GF), and the leaf physiological indices were measured and transcriptome sequencing was performed. The results showed that BF treatment significantly inhibited the height growth, leaf length, and leaf width of *P. qamdoensis*, while significantly increasing the thickness of leaf palisade tissue, upper epidermis, and the density of leaf structure. The GF treatment increased stomatal conductance (Gs) and intercellular CO_2_ concentration (Ci), while the BF treatment enhanced water use efficiency (WUE). Both BF and GF increased the contents of chlorophyll b (Chl b) and carotenoids (Car). BF treatment increased the content of total soluble sugars but decreased the contents of sucrose and starch. Transcriptome analysis revealed that under BF treatment, most genes in the sucrose and starch metabolism pathways were up-regulated, and the *AUX/IAA*, *GH3*, and *SAUR* genes in the auxin pathway also showed an up-regulated trend. In contrast, under GF treatment, most genes in the porphyrin and chlorophyll metabolic pathway were up-regulated, and most genes in the gibberellin pathway also showed up-regulation. Analysis of photoreceptor gene expression showed that GF treatment significantly up-regulated the expression of *HYH*, *COP1*, *CRY1*, *HY5*, and *PIF4* genes, while BF treatment had the opposite effect. These results provide a theoretical basis for revealing the evolutionary mechanisms underlying the adaptation of plants to different light environments.

## Simple Summary

To explore the growth mechanisms of plants in response to light environments, *Populus qamdoensis* cuttings were treated with transparent colorless film (WF), blue film (BF), and green film (GF), and the leaf physiological indices and transcriptome sequencing were examined in this study. The results indicated that different colored films differentially affect the growth and physiological characteristics of *P. qamdoensis* by regulating the expression of core metabolic and signal pathway genes mediated by photoreceptors, providing an important theoretical basis and practical guidance for improving seedling traits, enhancing photosynthetic efficiency, and stress resistance through light environment regulation technology in *P. qamdoensis* seedling cultivation.

## 1. Introduction

Plants are influenced by a variety of environmental factors throughout their ontogeny, with light being among the most pivotal [1]. Light is essential for regulating plant growth processes in addition to providing the energy needed for plant growth and development [2]. The light environment modulates plant morphogenesis via multiple dimensions, including photoperiod, light intensity, and light quality [3,4]. Light quality, in particular, stands out among these as a key regulator of plant growth and development [5]. During seedling cultivation, fluctuations in light quality can induce distinct variations in the plant growth process [6].

Plant morphogenesis is significantly impacted by various light qualities [7,8]. While increasing the amount of blue light contributes to improving stem diameter, enhancing the amount of red light can boost plant height, leaf area, and growth when red and blue light are combined [9,10]. Green light is a pivotal factor in plant growth, as it stimulates morphogenesis, cell division, and photosynthesis [11,12]. Studies have demonstrated that green light reduces the leaf angle of *Arabidopsis thaliana* while increasing leaf and petiole lengths [13]. When green light substitutes for a portion of red light under a red and blue light background while keeping the total light intensity unchanged, the fresh weight, dry weight, and leaf area of plants all exhibit significant increases [14].

Plants perceive fluctuations in light intensity, photoperiod, and light irradiation via photoreceptors, which function to sense, capture, and transduce light signals to downstream molecular targets [15,16]. Through diverse intracellular signal transduction cascades, photoreceptors modulate the expression of light-responsive genes, ultimately triggering physiological alterations at both cellular and organismal levels—enabling plants to adjust their growth and metabolism in response to dynamic light environments [17,18,19]. Research has shown that blue light, acting through cryptochrome 1 (CRY1), inhibits the degradation of DELLA proteins—core negative regulators in the gibberellin (GA) signaling pathway—thereby repressing the GA response during hypocotyl elongation [20]. Additionally, blue light-activated CRY1 competes with AGB1 for binding to HY5, a key transcription factor, thereby enhancing HY5’s biochemical activity in photomorphogenesis [21]. Furthermore, multiple photoreceptors, including phytochromes and cryptochromes, facilitate the accumulation of HY5 protein under distinct light conditions [22,23]. HY5 interacts with calmodulin CAM7, and this complex binds to the promoter region of the HY5 gene itself, forming a positive regulatory feedback loop by upregulating HY5 transcription, which further reinforces its role in photomorphogenic regulation [24].

*Populus* is used as a model plant for molecular biology research, including biotechnology and genetic engineering, owing to its excellent characteristics such as rapid growth, strong adaptability, and ease of asexual reproduction [25,26]. However, plants exhibit significant variability in their photosynthetic responses to light quality. While light-emitting diodes (LEDs) are widely used as light sources in most light quality regulation studies, their applicability in woody plant research and production is constrained by the long growth cycles and large stature of woody species, coupled with the high cost of LED applications. In contrast, adjusting light quality using colored films with similar light transmittance offers the advantages of simplicity and low cost. By deploying films of different colors to optimize the plant light environment, it is feasible to maximize photosynthetic efficiency, shorten growth cycles, and increase yields. Previous studies have applied colored films to regulate light quality for vegetables, flowers, and some woody plants, revealing that the effects of the same light quality component vary across different plants [27]. Research has shown that green film treatment significantly improves sugar content and photosynthetic parameters in *P. trinervis*, whereas blue film treatment markedly enhances its absolute growth and chlorophyll content [28]. Blue film treatment promotes the accumulation of photosynthetic pigments, sugar content, and photosynthetic performance of *P. szechuanica* var. *tibetica* seedlings, while green film treatment increases its photorespiration rate and CO_2_ compensation point [29]. These findings indicate that interspecific differences in genetic characteristics and light adaptation capabilities lead to distinct plant responses to light quality.

*Populus qamdoensis*, a species belonging to the section *Tacamahaca* of the genus *Populus* in the Salicaceae family, is primarily distributed in areas such as Qamdo, Tibet, at elevations ranging from 3400 to 3800 m [30,31]. As a native species in southwest China, it exhibits prominent traits including strong stress resistance, rapid growth, and excellent adaptability to the complex local geographical and climatic conditions [32]. However, current research on the photosynthetic physiological and biochemical changes, leaf tissue structure, and expression patterns of related genes in *P. qamdoensis* under different light quality conditions remains unclear, and the regulatory mechanism of light quality during its seedling cultivation and growth processes also needs to be elucidated. Therefore, this study employed annual cuttings of *P. qamdoensis* as materials, utilizing three distinct colored plastic films to simulate varying light quality conditions. Through systematic measurement of plant phenotypic growth traits, leaf anatomical structure, photosynthetic physiology, and biochemical indicators related to material metabolism, combined with transcriptome sequencing to screen differentially expressed genes (DEGs) by qRT-PCR. This study provides an in-depth analysis of the growth adaptation, physiological responses, and molecular regulatory mechanisms of *P. qamdoensis* cuttings under varying light qualities. It aims to establish a theoretical foundation for elucidating the evolutionary strategies of plant adaptation to light quality, while simultaneously offering scientific support for optimizing light environment regulation techniques in *P. qamdoensis* seedling propagation and cultivation management, thereby enhancing cultivation efficiency.

## 2. Materials and Methods

### 2.1. Plant Material and Light Treatment

*P. qamdoensis* were collected from a natural forest in Qamdo (97°01′17″ E, 31°11′44″ N), Tibet, China, and maintained via cutting propagation in Southwest Forestry University’s asexual nursery. Uniform, healthy one-year-old trunks were selected, and their middle segments (with plump buds) were cut into 25 cm scions, which were planted in 42 cm-diameter pots with a 1:2:1 substrate of red soil: charcoal soil: humus. After four months, the cuttings were transferred to a greenhouse and covered with 0.1 mm-thick translucent colorless film (WF), blue (BF), and green (GF) film, each treatment replicated three times. The plastic film treatment is maintained for 60 days, with the film being replaced approximately every 20 days to ensure consistent treatment efficacy.

### 2.2. Determination of Phenotypic Indicators and Leaf Tissue Structure Parameters

Seedling height (main branch length) and ground diameter (2 cm above main branch base) of *P. qamdoensis* were measured with a tape measure and Vernier caliper, respectively. Each treatment included three biological replicates, with the average of three seedlings from a single asexual line considered one replicate.

To analyze leaf tissue structure, one plant was randomly selected from each asexual line per treatment. The 5th leaf from the top of the main stem was sampled, and a 2 cm × 2 cm section (centered over the main vein) was excised. The leaf section was placed in FAA fixative and fixed after vacuuming. Leaf tissue dissection was performed following the method described by Zhou et al. [33]. Cross-sections of leaf blades and leaf veins were observed using an Olympus BH-2 optical microscope equipped with a Motic micro-imaging system. Tissue structural parameters were measured using Motic DigiLab abll 3.0 software, including leaf thickness (LT), the thickness of the upper (UE) and lower epidermal (LE) cells, the palisade tissue thickness (PT), and the spongy tissue thickness (STT). Three seedlings were selected from each asexual line, and 10 fields of view were measured per sample to calculate the mean value. The cell-to-tense ratio (CTR), sponge ratio (SR), and palisade tissue and sponge tissue ratio (PS) were computed by the following formula: CTR = (PT/LT) × 100%; SR = (STT/LT) × 100%; palisade tissue and sponge tissue ratio = PT/ST.

### 2.3. Measurement of Photosynthetic Characteristics Under Different Treatments

The photosynthetic gas exchange parameters of *P. qamdoensis* seedlings under different treatments were determined using the LI-6800 Portable Photosynthesis System. For sample selection, the 5th to 7th leaves, counted from top to bottom at the apex of the main stem of each *P. qamdoensis* seedling, were designated as the test leaves. Determinations were carried out at 2 h intervals from 8:00 to 18:00. The core parameters measured included the net photosynthetic rate (Pn), the transpiration rate (Tr), intercellular CO_2_ concentration (Ci), stomatal conductance (Gs), and water use efficiency (WUE) (WUE = Pn/Tr). Measure 3 leaves per plant, with 3 plants per clonal line serving as 3 biological replicates.

### 2.4. Determination of Photosynthetic Pigments and Sugar Content

Fresh leaves (5th~7th leaves from the top of the main stem downward) were collected for photosynthetic pigments determination. Pigments were extracted using the ethanol grinding-extraction method. A 721 visible spectrophotometer (Shanghai Jinghua Technology Instrument Co., Ltd., Shanghai, China) was used to measure the absorbance values of the extracted solution at wavelengths of 665 nm (A_665_), 649 nm (A_649_), and 470 nm (A_470_), respectively. For each sample, three technical measurements were conducted, and their average value was regarded as one replicate. Each treatment included three biological replicates. The contents of chlorophyll a (Chl a), chlorophyll b (Chl b), and carotenoids (Car) were calculated using the formulas described by Cai et al. [34].

Chla (mg·g^−1^ FW) = ((13.95A665 − 6.88A649) × Total amount of extract) ÷ (Sample fresh weight × 1000)

Chlb (mg·g^−1^ FW) = ((24.96A649 − 7.32A665) × Total amount of extract) ÷ (Sample fresh weight × 1000)

Car (mg·g^−1^ FW) = ((1000A470 + 3.27 × (13.95A665 − 6.88A649) + 104 × (24.96A649 − 7.32A665)) ÷ (229 × Total amount of extract) ÷ (Sample fresh weight × 1000)

The anthrone-H_2_SO_4_ method and the 3,5-dinitrosalicylic acid method [35] were employed to prepare extracts of total soluble sugar, reducing sugar and starch extracts, respectively, for sugar content determination. A visible spectrophotometer was used to measure the absorbance values of the extracts, and the contents of leaf sucrose and starch were calculated following the corresponding methods described by Gao et al. [35]. For each sample, three technical measurements were performed, and their average value was taken as one replicate; each treatment included three biological replicates.

Reducing sugar content (%) = (C × Vt) ÷ (W × Vs) × 100%

Soluble sugar content (%) = (C × Vt × n) ÷ (W × Vs × 1000) × 100%

Starch content (%) = (C × Vt × 0.9) ÷ (W × Vs × 1000) × 100%

Sucrose content (%) = (Soluble sugar content—Reducing sugar content) × 0.95

C: glucose content in the sample calculated using the glucose standard curve (mg); Vt: total volume of sample extract (mL); Vs: volume of the sample used in the determination (mL); W: dry weight of the sample (mg); n: dilution times; 1000: conversion coefficient; 0.9: coefficient of glucose conversion to starch.

### 2.5. Transcriptome Data Analysis

After 60 days of irradiation treatment, leaves were sampled from three plants per treatment. Total RNA was extracted using the Plant RNA Kit (Omega, Beijing, China). RNA quantity was determined by measuring the A_260_/A_280_ ratio with a K5800C spectrophotometer (KAIAO, Beijing, China), and RNA integrity was assessed using an Agilent 2100 system (Agilent Technologies, Santa Clara, CA, USA). High-quality RNA samples were used to construct complementary DNA (cDNA) libraries. Each library generated approximately 6 gigabases (Gb) of raw sequencing data on the Illumina platform. The filtered reads were compared to the *P. yunnanensis* reference genome (PRJNA886471).

For RNA-Seq data analysis, *p*-values were adjusted using the Benjamini–Hochberg method to control the false discovery rate (FDR). Differentially expressed genes (DEGs) were identified under the screening criteria of Fold Change (FC) ≥ 1.5 and adjusted *p*-value < 0.01. Gene Ontology (GO) enrichment analysis was performed on the DEGs, which were then mapped to GO terms in the GO database (http://www.geneontology.org/ (13 August 2021)). To analyze pathways significantly associated with DEGs, the DEGs were blasted against the Kyoto Encyclopedia of Genes and Genomes (KEGG) database (https://www.kegg.jp/ (13 August 2021)) using the same statistical method. After counting the number of genes assigned to each GO term or KEGG pathway, significantly enriched pathways were identified via a hypergeometric test.

### 2.6. Prediction of Light-Responsive Protein Interaction Networks

Interaction relationships among light-responsive proteins were obtained using STRING software (https://string-db.org/ (19 May 2025)), followed by further analysis with Cytoscape 3.7.0 software to assess and predict the interaction information of these proteins.

### 2.7. Quantitative Real-Time PCR (qRT-PCR) Analysis

Based on the results of pathway-specific gene annotation, DEGs that were closely associated with KEGG pathways and exhibited significant fold changes in expression were selected for qRT-PCR validation. The qualified RNA was used for 1st strand cDNA synthesis using Hifair^®^ III Reverse Transcriptase (YEASEN, Shanghai, China). The qRT-PCR primer pairs were designed based on the coding sequence (CDS) of the selected DEGs via NCBI primer BLAST 2.16.0 (Table S1) and synthesized by Sangon Biotech Co., Ltd. (Shanghai, China). The PED1 gene was the internal reference gene [36]. qRT-PCR reactions were performed using Hieff UNICON^®^ Universal Blue qPCR SYBR Green Master Mix (YEASEN, Shanghai, China) on a LightCycler^®^ 96 Real-Time PCR Detection System (Roche, Hercules, Switzerland). Each sample was analyzed with three biological replicates and three technical replicates per biological replicate. The relative expression levels of the target genes were calculated using the 2^−∆∆Ct^ method [37].

### 2.8. Statistical Analysis

All data were analyzed using SPSS 26.0 software and expressed as the mean ± standard deviation (SD) of three replicates. Duncan’s multiple range test was used to analyze differences among multiple means, while the least significant difference (LSD) test was employed for pairwise comparisons between groups (*p* < 0.05). The diagram was generated using Origin 8.5 and GraphPad Prism 8.0 software.

## 3. Results

### 3.1. Ratio Analysis of Transmission Spectra in the Shed

**Figure 1:**
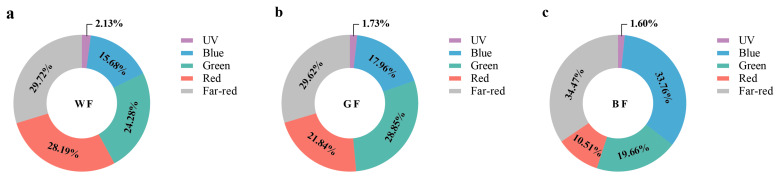
Transmission spectral ratios of different color films. (**a**–**c**) are pie charts showing the proportion distribution of spectral transmittance under WF, GF, and BF conditions, respectively. Among them, UV stands for ultraviolet light, Blue for blue light, Red for red light, Green for green light, and Far-red for far-red light. The percentage of each color block indicates the proportion of the corresponding spectrum under that lighting condition.

Varied colored films have varied light-transmission properties (Figure 1). Compared to BF and GF, WF transmits a greater percentage of red and UV light. In comparison to GF and WF, BF transmits a greater percentage of blue and far-red light. Compared to BF and WF, GF transmits more green light, and it transmits more red light than BF. The BF and GF’s transmitted wavelengths clearly match the wavelengths of their corresponding colors. Of them, far-red light can be transmitted more easily through BF, whereas red light can be transmitted more easily through WF.

### 3.2. Effects of Different Film Treatment on P. qamdoensis Growth

**Figure 2:**
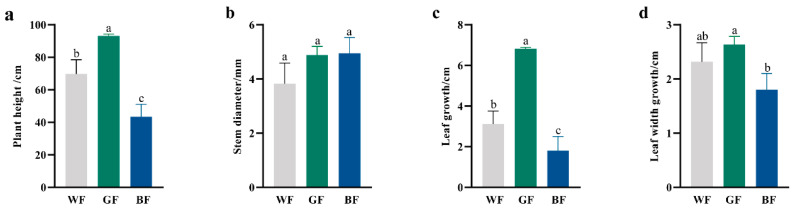
Comparison of growth of *P. qamdoensis* under different film treatments. (**a**–**d**) are bar charts showing the plant height, stem diameter, leaf length, and leaf width of *P. qamdoensis* under WF, GF, and BF conditions, respectively. Different lowercase letters (a, b, c) indicate significant differences between groups at the *p* < 0.05 level.

Following 60 days of colored film treatments, significant differences were observed in the growth of *P. qamdoensis*. GF treatment exerted a substantial effect on the vertical growth and leaf expansion of *P. qamdoensis*, seedling height (Figure 2a), leaf length (Figure 2c), and leaf width (Figure 2d) under GF were significantly greater than those under WF and BF treatments. However, there was no significant difference in the ground diameter (Figure 2b) of *P. qamdoensis* among the different film treatments, indicating that film type had a negligible impact on this trait. In summary, GF treatment strongly promoted seedling height and leaf development in *P. qamdoensis*, while its effect on ground diameter was not significant, further confirming the growth-promoting role of GF in key aboveground growth traits.

### 3.3. Effects of Different Film Treatment on the Leaf Tissue Structure of P. qamdoensis

**Figure 3:**
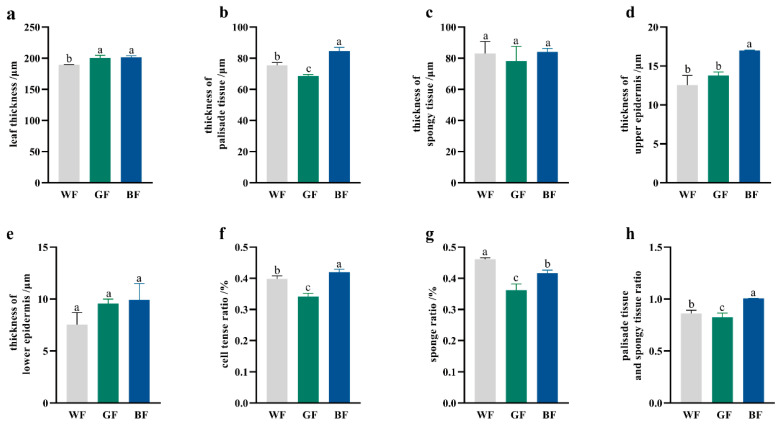
Structural parameters of leaves of *P. qamdoensis* under different film treatments. (**a**–**h**) are bar charts showing the leaf thickness, palisade tissue thickness, spongy tissue thickness, upper epidermal thickness, lower epidermal thickness, cell tense ratio, sponge ratio, and palisade tissue and spongy tissue ratio of *P. qamdoensis* under WF, GF, and BF conditions, respectively. Different lowercase letters (a, b, c) indicate significant differences between groups at the *p* < 0.05 level.

Under different treatments, *P. qamdoensis* exhibited significant differences in leaf tissue structural traits. Among the three colored film treatments, leaf thickness of *P. qamdoensis* differed significantly (Figure 3a), leaves under WF were significantly thinner than those under BF and GF, with the greatest thickness observed under BF, followed by GF, though the difference between BF and GF was not statistically significant. Additionally, BF treatment consistently yielded higher values than WF and GF treatments across multiple traits, including palisade tissue thickness (Figure 3b), spongy tissue thickness (Figure 3c), upper (Figure 3d) and lower epidermal thickness (Figure 3e), cell-to-tense ratio (Figure 3f), and sponge tissue ratio (Figure 3h).

### 3.4. Effects of Different Colored Films on Photosynthetic Characteristics of P. qamdoensis

**Figure 4:**
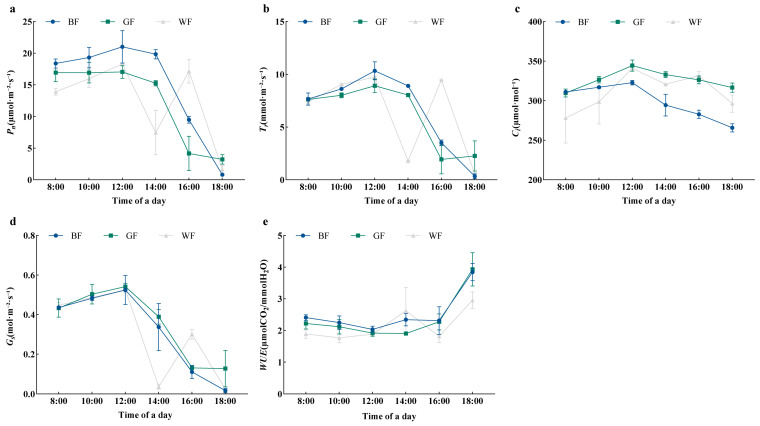
Diurnal variation in photosynthetic gas exchange parameters of *P. qamdoensis* under different film treatments. (**a**–**e**) are line graphs showing the dynamic changes in Pn, Tr, Ci, Gs, and WUE of *P. qamdoensis* under BF, GF, and WF conditions throughout a day (8:00–18:00), respectively. Different colored lines represent different treatment conditions (BF, GF, WF).

*P. qamdoensis* exhibited consistent diurnal fluctuation trends in Pn, Tr, Ci, and Gs under various film treatments (Figure 4a–d). With two peaks at 12:00 and 16:00, respectively, the diurnal fluctuations of these indicators under WF treatment showed a ‘double-peak’ curve, and at 14:00, there was a clear ‘midday photosynthetic depression’ phenomenon. The peaks of Pn under both treatments reached the daily maximum at 12:00, and the diurnal fluctuations of all indicators under BF and GF treatments displayed a ‘single-peak’ curve. Furthermore, Pn was significantly higher in the BF treatment than in the WF treatment, while the GF treatment exhibited lower Pn than the WF, though the difference was not statistically significant, according to the daily average values of photosynthetic parameters under the various treatments. While *Tr* was much lower in the GF treatment than in the WF treatment, Gs and Ci were significantly higher in the GF treatment. For Tr and Gs, there was no discernible change between BF and WF; however, Ci was considerably lower in the BF therapy than in the WF treatment. The sequence of the daily average *WUE* was BF > GF > WF, with BF and WF differing significantly (Figure 4e).

### 3.5. Effects of Different Colored Films on Photosynthetic Pigments and Sugar Content of P. qamdoensis

**Figure 5:**
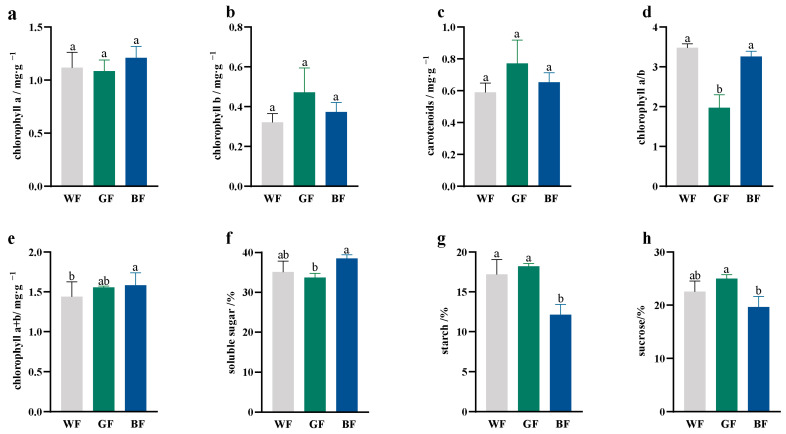
Photosynthetic pigment and sugar contents in leaves of *P. qamdoensis* under different film treatments. (**a**–**h**) are bar charts showing the chl a content, chl b content, car content, chl a/b ratio, total chl content, soluble sugar content, starch content, and sucrose content of *P. qamdoensis* under WF, GF, and BF conditions, respectively. Different lowercase letters (a, b) indicate significant differences between groups (*p* < 0.05).

Leaves of *P. qamdoensis* under BF treatment exhibited higher contents of Chla (Figure 5a), Chlb (Figure 5b), Car (Figure 5c), Chla + b (Figure 5e), and total soluble sugar compared to those under WF treatment; however, these differences did not reach statistical significance. Notably, the starch and sucrose contents (Figure 5g,h) in the BF treatment were significantly lower than in the WF treatment. Under GF treatment, *P. qamdoensis* showed decreased contents of Chl a, Chl a/b ratio (Figure 5d), and total soluble sugar, but increased contents of Chl b, Car, Chl a + b, starch, and sucrose. It showed that the BF treatment promoted the accumulation of total soluble sugar (Figure 5f) and increased the contents of Chl a and Chl b in the *P. qamdoensis* leaves, while GF treatment not only enhances the accumulation of starch and sucrose but also increases the contents of Car and Chl b.

### 3.6. Correlation Analysis

**Figure 6:**
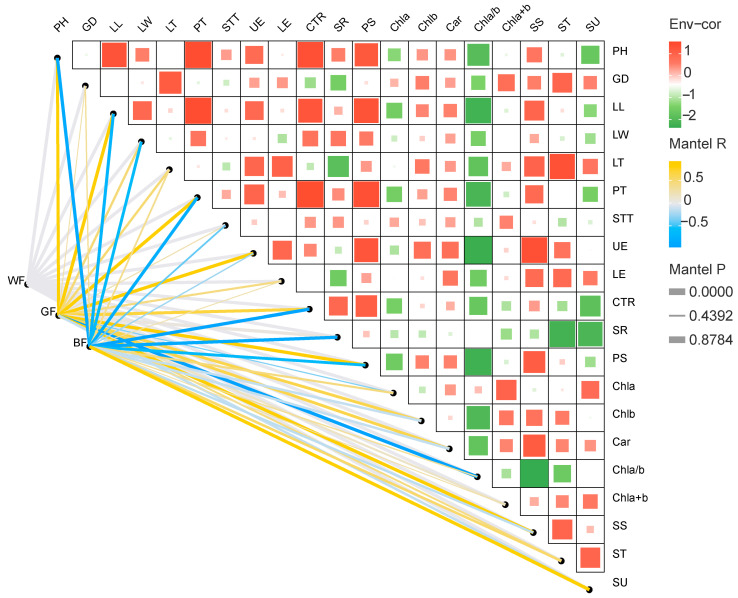
Correlation between different film treatments and observation indexes of *P. qamdoensis*. PH: plant height, GD: ground diameter, LL: leaf length, LW: leaf width, LT: leaf thickness, PT: palisade tissue thickness, STT: spongy tissue thickness, UE: upper epidermal thickness, LE: lower epidermal thickness, CTR: cell tense ratio, SR: sponge ratio, PS: palisade tissue and sponge tissue ratio, Chla: chlorophyll a, Chlb: chlorophyll b, Car: carotenoids, SS: Soluble sugar content, ST: starch content, SU: sucrose content. In the heatmap, the horizontal and vertical axes represent various indicators that interact with growth physiology, respectively. The color of the color block in each grid of the heatmap indicates the positive or negative correlation coefficient between growth and physiological indicators, and the size of the color block represents the absolute value of the correlation coefficient. The lines represent the one-to-one correlation between light environment data and growth-physiological indicator data. The thickness of the lines indicates the strength of the correlation, and the color of the lines indicates the significance level.

Correlation analysis revealed that most growth and physiological traits of *P. qamdoensis* seedlings were significantly positively correlated with GF treatment, whereas the opposite trend was observed for BF treatment (Figure 6). Specifically, growth indices and leaf tissue structural traits showed positive correlations with GF; in contrast, plant height (PH), leaf length (LL), PT, and UE exhibited highly significant negative correlations with BF. Among photosynthetic pigment and sugar content parameters, both GF and BF were positively correlated with total chlorophyll and ST contents, while their correlations with other parameters (e.g., chlorophyll a/b ratio, total soluble sugar) showed opposing patterns. These results clearly indicate that *P. qamdoensis* exhibits distinct response patterns to different light quality conditions.

### 3.7. Analysis of DEGs Following the BF and GF Treatments by RNA-Seq

**Figure 7:**
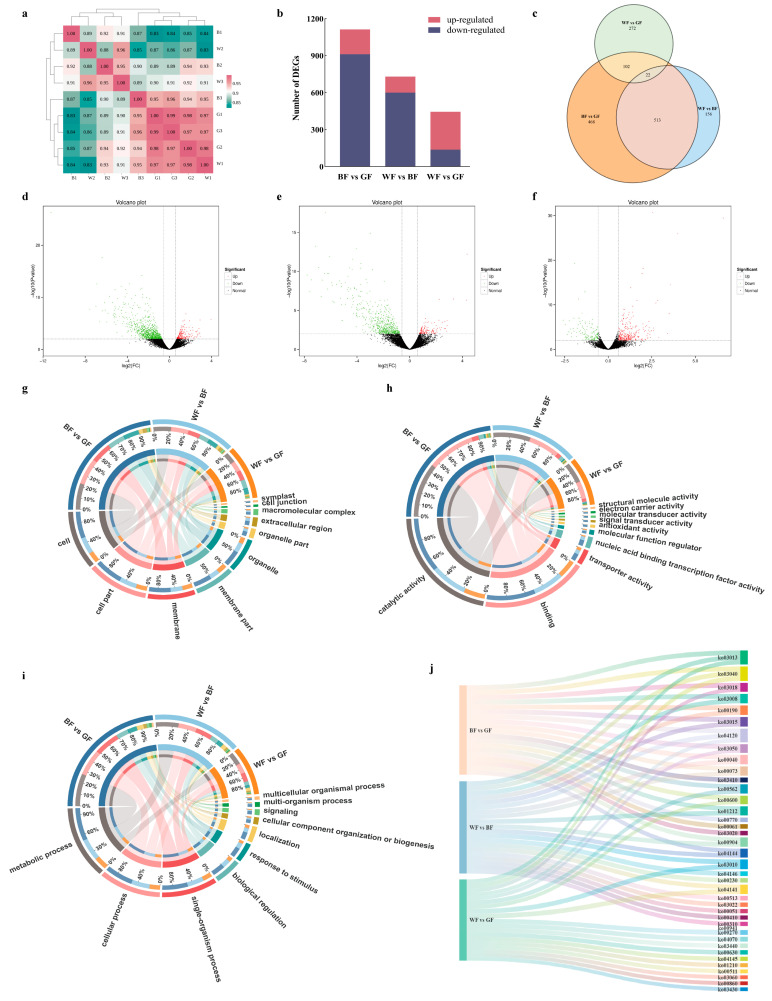
Transcriptome analysis of *P. qamdoensis* under three different color film treatments. (**a**) Sample correlation analysis. (**b**) Number of up- and down-regulated DEGs. (**c**) Venn diagram. (**d**–**f**) Each point in the volcano plot represents a gene. The abscissa (x-axis) denotes the logarithmic value of the fold change in gene expression between two samples, while the ordinate (y-axis) represents the negative logarithmic value of the statistical significance of gene expression changes. In the plot, green points indicate down-regulated DEGs, red points signify up-regulated DEGs, and black points represent non-DEGs. (**g**–**i**) GO enrichment analysis; (**g**) cellular component entries; (**h**) molecular function entries; (**i**) biological process entries. The outer color strip in the upper part shows the comparison groups, and the inner circle represents the composition of the comparison group. The outer color strip in the lower part shows the composition, and the inner circle indicates the abundance distribution of the composition among the comparison groups. The thickness of the chords in the middle part reflects the abundance level of the composition in each comparison group. (**j**) Analysis of KEGG pathway top 20 enrichment in three comparison groups. In the Sankey diagram, the three color bars on the left represent the three comparison groups, and the color bars on the right represent KEGG pathways. The flow direction of the bands indicates the correlation between data, and the width of the bands reflects the strength of the correlation between data.

Transcriptome sequencing was performed on leaf samples subjected to different colored film treatments. Sequencing quality and biological replicate correlation were evaluated using Q30, GC content, and Pearson’s correlation coefficients (Figure 7a, Table S2), with results confirming high-quality sequencing data and strong consistency among biological replicates. Among the three comparison groups, BF and GF exhibited the largest number of DEGs, totaling 1113 genes, with 202 up-regulated and 911 down-regulated genes. Compared to the WF group, the BF group contains 308 up-regulated genes and 137 down-regulated genes, while the GF group had 130 up-regulated genes and 600 down-regulated genes (Figure 7b–f).

Further GO enrichment analysis of DEGs in each comparison group revealed that enriched genes were primarily concentrated in cellular component terms, with the most abundant DEGs associated with “cell,” “membrane,” and “cell part” (Figure 7g; Table S3). In molecular function terms, DEGs related to “catalytic activity” and “binding” were most prevalent (Figure 7h). For biological processes, DEGs were predominantly involved in “metabolic process,” “cellular process,” and “single-organism process” (Figure 7i). Analysis of the top 20 KEGG pathways enriched across all comparison groups identified “Plant-pathogen interaction” (ko00592), “Plant hormone signal transduction” (ko00040), “MAPK signaling pathway—plant” (ko00511), and “Starch and sucrose metabolism” (ko00860) as the pathways with the highest number of DEGs. In the WF vs. BF group, the most significant enrichment factors were observed for “Amino sugar and nucleotide sugar metabolism” (ko00640), “Flavonoid biosynthesis” (ko04120), and “Phenylalanine, tyrosine and tryptophan biosynthesis” (ko00970). In the WF vs. GF group, pathways with the most significant enrichment factors included “Plant circadian rhythm” (ko00903), “Flavonoid biosynthesis” (ko04120), “Photosynthesis” (ko03013), and “Starch and sucrose metabolism” (ko00860) (Figure 7j; Table S4).

### 3.8. Pathway Analysis of P. qamdoensis in Response to Different Colored Film Treatments

**Figure 8:**
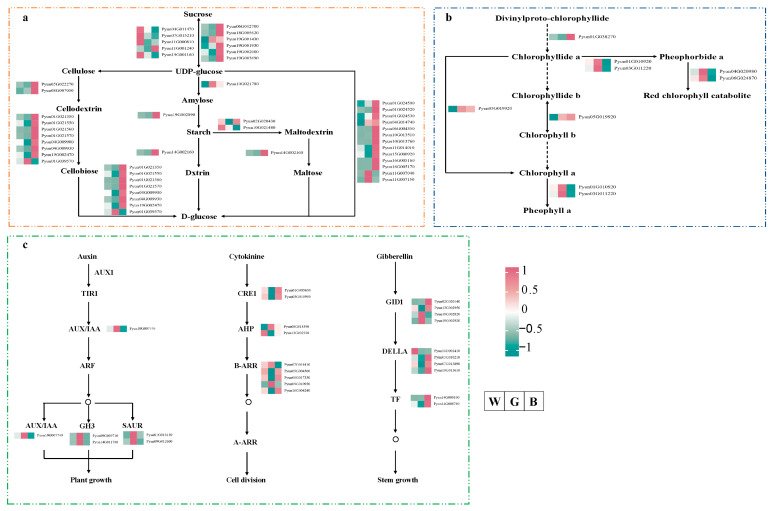
Pathway analysis of *P. qamdoensis* under different color film treatments. (**a**) starch and sucrose metabolism pathway. (**b**) porphyrin and chlorophyll metabolism pathway. (**c**) plant hormone signal transduction pathway. The heatmaps show FPKM values of the DEGs. The rows of the heatmap represent different DEGs, the columns represent gene expression levels under different treatment conditions, and the color scale denotes the expression levels, which are in the order of WF, GF, and BF from left to right.

To dissect the growth regulatory network of *P. qamdoensis* under different colored film treatments and deeply explore the underlying mechanisms of physiological phenotype changes, this study further analyzed the starch and sucrose metabolism, porphyrin and chlorophyll metabolism, and plant hormone signal transduction pathway (Figure 8; Table S5). The metabolic pathway for starch and sucrose in *P. qamdoensis* was examined while the plant was treated with various colored films. The results showed that a total of 39 DEGs encoding 8 types of enzymes were identified. Most DEGs were up-regulated under BF treatment, whereas the opposite expression trend was observed under GF treatment (Figure 8a).

Analysis of the porphyrin and chlorophyll metabolic pathways revealed that a total of 6 DEGs encoding 4 enzymes were identified. Under GF treatment, most of these genes were up-regulated, while BF treatment induced the opposite expression pattern (Figure 8b). DEGs linked to plant growth in plant hormone signal transduction pathways were analyzed. The findings demonstrated that AUX/IAA, GH3, and SAUR gene expression levels in the auxin pathway were all up-regulated in response to BF treatment and down-regulated in response to GF treatment. 9 DEGs encoding three different types of enzymes were found to be involved in the cytokinin pathway. These genes exhibited both up- and down-regulated trends when exposed to various colored films, but the majority of them were up-regulated when exposed to GF. The majority of the genes resulting in GID1, DELLA, and TF genes in the gibberellin pathway expressed more when exposed to GF than when exposed to WF or BF (Figure 8c).

### 3.9. Expression Patterns of Light Signaling-Related Transcription Factors (TFs)

**Figure 9:**
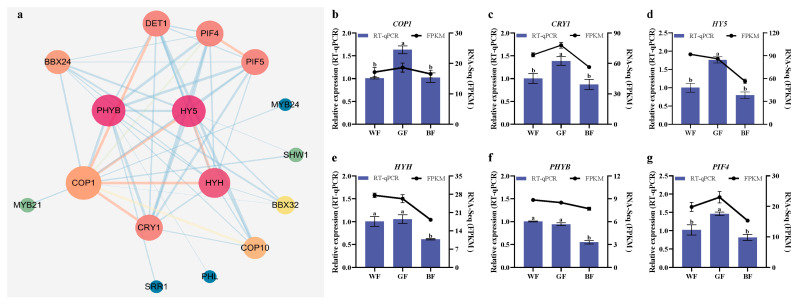
Analysis of expression patterns of light-responsive TFs. (**a**) Co-expression regulatory network of light-responsive TFs. Each node in the regulatory network represents a protein, where the size of the node is proportional to its correlation with light response, and the color of the node corresponds to its size. Colors tending towards red indicate that the corresponding node is a “core hub” within the network, where its interactions perform crucial regulatory or connecting functions across the entire network. Colors tending towards yellow-orange denote nodes classified as “intermediate hubs”, possessing a certain capacity for interaction. Colors tending towards blue-green signify that the node exhibits sparse connectivity within the network, with its corresponding interactions falling within the category of “secondary interactions”. The lines indicate interactions between proteins, with thicker lines representing stronger interactions. (**b**–**g**) Validated qRT-PCR results for the six genes. The left Y-axis denotes relative gene expression levels, while the right Y-axis represents transcriptome FPKM values. Data are presented as mean ± SD (n = 3). One-way analysis of variance was employed to compare gene expression differences under WF, GF, and BF treatment conditions. Different letters indicate significant differences in gene expression levels across the three treatments (*p* ≤ 0.05).

To explore the mechanism by which light quality regulates the growth of *P. qamdoensis*, this study analyzed the expression patterns of light-responsive TFs. A co-expression regulatory network of light-responsive TFs was constructed, revealing a total of 16 proteins in the network (Figure 9a). Among these, *PHYB*, *HY5*, *HYH*, *COP1*, *CRY1*, and *PIF4* showed relatively close interactions with other TFs (evidenced by a higher number of connecting edges in the network), indicating that these TFs may serve as key response nodes in the light quality regulatory pathway. Further analysis of expression levels demonstrated that the expression of *COP1*, *CRY1*, *HY5*, and *PIF4* was significantly up-regulated under GF treatment. In contrast, *HYH*, *PHYB*, and *PIF4* exhibited a significant down-regulation trend under BF treatment (Figure 9b–g). Notably, the expression trends of these genes were consistent with the transcriptome data, confirming the reliability of the expression pattern analysis.

## 4. Discussion

### 4.1. Effects of Different Colored Film Treatments on the Growth and Development of P. qamdoensis

Comparative analysis of *P. qamdoensis* cutting growth under different colored film treatments revealed distinct growth performance between BF and GF treatments. BF significantly inhibited seedling height, leaf length, and leaf width, whereas GF significantly promoted these growth indicators. Notably, neither treatment affected radial growth (ground diameter) significantly. This aligns with findings by Trouwborst et al. [38] and Paradiso et al. [39], who reported that blue light can suppress seedling growth.

**Figure 10:**
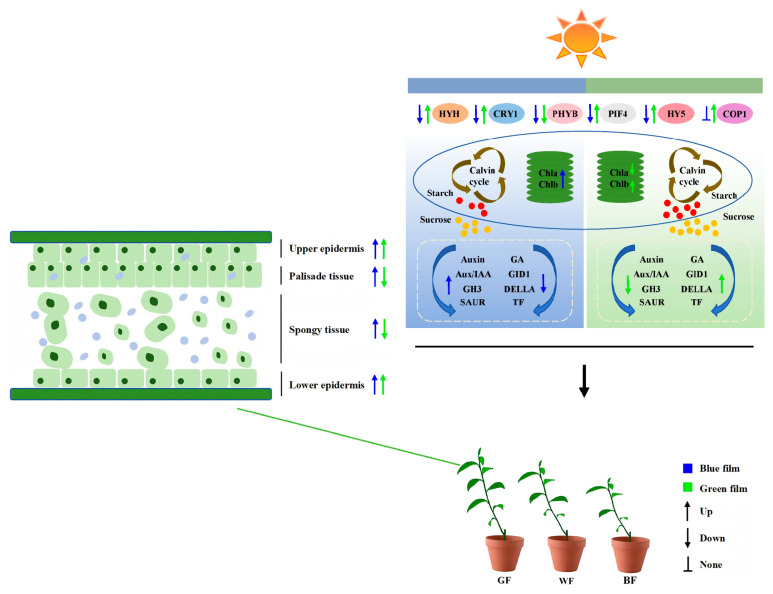
Model of the effects of BF and GF treatments on the growth of *P. qamdoensis*. Under BF treatment, the expression of photoreceptor genes *(HYH*, *CRY1*, *PHYB*, *PIF4*, and *HY5*) decreases, while genes in the porphyrin and chlorophyll metabolism pathway are up-regulated, promoting an increase in the contents of Chl a and Chl b. In the sucrose and starch metabolism pathways, most genes show an up-regulated trend, which accelerates the decomposition of starch and sucrose, resulting in a decrease in their contents. In the hormone signaling pathway, the negative regulators (*AUX/IAA*, *GH3*) in the auxin pathway are up-regulated, inhibiting auxin-mediated cell elongation; at the same time, the expression of genes in the gibberellin pathway is suppressed, further weakening the driving force for longitudinal growth. These changes are physiological responses of plants to adapt to strong light exposure, such as increasing the thickness of mesophyll cells, which collectively lead to the inhibition of *P. qamdoensis* height growth. In contrast, the GF treatment shows an opposite trend: the expression of photoreceptor genes is enhanced, the expression patterns of genes in the porphyrin and chlorophyll metabolism and sucrose and starch metabolism pathways are opposite to those under BF treatment, and the growth-promoting genes in the hormone signaling pathways (such as genes in the gibberellin pathway) are up-regulated. Ultimately, through physiological regulation, adapting to low-light environments, the growth of seedlings is promoted.

Environmental factors strongly influence plant photosynthetic traits, with leaf tissue structural modifications often mediating changes in photosynthetic function [40,41]. Under GF treatment, the stomatal conductance of *P. qamdoensis* was significantly elevated, which was substantiated by notably higher values of Tr, Gs, and Ci relative to the WF treatment. This result is closely correlated with the increased thickness of the lower epidermis in *P. qamdoensis* subjected to GF treatment. Previous studies have demonstrated that a large number of stomata are distributed on the leaf lower epidermis, and the enhancement of leaf gas exchange capacity can be achieved by increasing the thickness of this tissue [42,43]. In this study, BF treatment significantly increased palisade tissue thickness, upper epidermis thickness, tissue structure compactness, and palisade-spongy ratio, whereas GF treatment induced the opposite trends (Figure 10). This indicates that *P. qamdoensis* is adapted to the relatively high proportion of short-wavelength light in its native environment. Under BF conditions, the species appears to reduce resource allocation to stem elongation and leaf area expansion, instead prioritizing enhancements to leaf photosynthetic structures (palisade tissue) and protective structures (upper epidermis). This adaptive strategy likely ensures survival and reproduction by improving photosynthetic efficiency and stress resistance. Conversely, in the GF environment, due to the low absorption ratio of green light in photosynthetically active radiation, the plants perceive insufficient photosynthetically active light, which triggers a ‘low-light adaptation strategy’-prioritizing the expansion of light-capturing range (increasing plant height, leaf length, and leaf width) to compensate for the insufficient photosynthetic efficiency through increased area. These findings are consistent with those of Dörken et al. [44] and Carriquí et al. [45], which indicate that characteristics such as well-developed palisade tissue, increased tissue structural compactness, and a higher palisade-to-spongy ratio are adaptive manifestations of plants to high-light habitats.

### 4.2. Specific Response Characteristics of Photosynthetic Pigments in P. qamdoensis to Different Light Qualities

Light quality changes significantly alter plant photosynthetic traits by regulating chlorophyll light absorption, thereby influencing light energy utilization efficiency [46,47]. The findings of this study showed that BF treatment significantly facilitated the accumulation of Chl a and Chl b in the leaves of *P. qamdoensis*, while GF treatment primarily increased the contents of Car and Chl b in the leaves. This result is consistent with the research finding that BF can promote the chlorophyll content in *Anoectochilus roxburghii* [48]. Further analysis of the porphyrin and chlorophyll metabolism pathway revealed that the majority of genes within this pathway exhibited an up-regulated expression trend under GF treatment, indicating that GF treatment may enhance the biosynthesis capacity of Chl b and Car by activating the transcription level of genes related to the porphyrin and chlorophyll metabolism pathway, and ultimately help *P. qamdoensis* adapt to the specific light quality environment mediated by GF by regulating the composition ratio of leaf photosynthetic pigments.

### 4.3. Association Between Metabolic Gene Expression and Photosynthate Allocation in P. qamdoensis Under BF and GF Treatments

Light quality influences soluble sugar content in plant leaves by inducing photoreceptors, regulating the activity of sugar-metabolizing enzymes, and modifying the efficiency of carbohydrate absorption in leaves [49,50]. The results of this study showed that although BF treatment significantly reduced the accumulation of sucrose and starch in the leaves of *P. qamdoensis* (Figure 10). It is speculated that the core reason is that BF treatment accelerated the translocation of photosynthates from leaves to various sink organs, resulting in reduced retention of the two substances in leaves, which is consistent with the trend observed in lettuce-related studies regarding the effect of light quality on photosynthate allocation [51]. In contrast, GF treatment significantly increased both sucrose and starch contents in leaves (Figure 10), indicating that GF may adapt to the light environment changes it mediates by slowing down the export rate of photosynthates or enhancing the carbon storage capacity of leaves. This result is consistent with the conclusions of similar studies on grape plantlets [52].

Further analysis of the sucrose and starch metabolism pathway revealed that most related genes in this pathway were up-regulated under BF treatment, while the contents of starch and sucrose in leaves showed a downward trend; GF treatment exhibited the opposite characteristics of down-regulated gene expression and increased product content. These results further reveal that BF treatment accelerates the decomposition, transformation, and transport of photosynthates to energy-consuming organs by up-regulating the expression of related genes, ultimately leading to reduced leaf accumulation. In contrast, in the low-light environment mediated by GF, *P. qamdoensis* shows down-regulated expression of related genes due to insufficient adaptability, which in turn inhibits the decomposition of photosynthates, and maintains normal life activities by increasing the accumulation of products in leaves.

### 4.4. Expression Characteristics of Hormone Signaling Pathway Genes in P. qamdoensis Under BF and GF Treatments

Light signals affect plant growth and development by regulating hormone levels and signal transduction processes in plant cells [53]. The results of this study showed that under BF treatment, the expression levels of *AUX/IAA*, *GH3*, and *SAUR* genes in the auxin signaling pathway were significantly down-regulated; whereas the *CRE1*, *AHP*, and *B-ARR* genes in the cytokinin pathway exhibited a fluctuating expression pattern under different film treatments; GF treatment significantly up-regulated the expression levels of most genes in the gibberellin pathway (Figure 10). This result indicates that GF treatment may promote cell elongation by enhancing the expression of genes in the gibberellin pathway, and may simultaneously indirectly weaken the inhibitory effect of the auxin pathway, prompting the gibberellin and auxin pathways to act synergistically to jointly promote stem elongation and leaf expansion, thereby endowing plants with growth advantages. In contrast, BF treatment inhibits cell elongation by down-regulating relevant genes in the auxin pathway, and this, combined with the low expression state of the gibberellin pathway, collectively leads to restricted plant growth. This result is consistent with the findings of studies on light quality regulation of *Cucumis sativus* growth [54] and light quality effects on *Picea abies* seedlings [55], suggesting that the growth response of *P. qamdoensis* to light quality may depend on the synergistic regulatory mechanism of gibberellins and auxins.

### 4.5. Characteristics and Adaptive Strategies of Light Signal Gene Expression in P. qamdoensis Regulated by Different Light Qualities

Different photoreceptors can trigger a series of biochemical reactions by perceiving and absorbing light energy of different wavelength bands, and play a role in plant morphogenesis, photosynthetic physiology, and metabolic physiology [56,57,58]. The results of this study showed that GF treatment significantly up-regulated the expression of *COP1*, *CRY1*, *HY5*, and *PIF4* genes in *P. qamdoensis* (Figure 10). As a positive regulator of light signal transduction, the increased expression level of *HY5* is usually associated with enhanced adaptability of plants to low-light environments, and can promote the synthesis of chlorophyll and the expression of photosynthesis-related genes [59,60]. However, as a negative regulator of *HY5*, the up-regulation of *COP1* under GF treatment may be a compensatory response of plants to maintain signal balance, which is similar to the research result that *COP1* and *HY5* are co-upregulated to fine-tune light response under low-light environments [61]. Furthermore, BF treatment significantly down-regulated the expression levels of *HYH*, *PHYB*, and *PIF4* genes in *P. qamdoensis* (Figure 10). Previous studies have indicated that high-intensity blue light inhibits the activity and gene expression of *PHYB*; the suppression of *PHYB* function would then weaken its transcriptional inhibitory effect on downstream PIF4, which should theoretically lead to an up-regulation of *PIF4* expression [62]. However, in this study, *PIF4* showed a significant down-regulation under BF treatment, which is distinctly different from the aforementioned blue light regulatory pattern. It is hypothesized that this phenomenon may be related to the woody plant characteristics of *P. qamdoensis*: the expression of *PIF4* in this species may not be regulated solely by *PHYB*, but rather mediated by a synergistic regulatory mechanism involving both *CRY1* and *PHYB*.

In summary, the differential regulatory effects of BF and GF treatments on the expression of key genes in the light signal transduction pathway of *P. qamdoensis* (such as *HYH*, *PHYB*, and *PIF4*) suggest that different light qualities may shape the plant’s strategy for adapting to specific light environments by precisely regulating the expression patterns of photoreceptors and core genes in downstream signaling pathways.

## 5. Conclusions

This study investigated the effects of WF, BF, and GF treatments on *P. qamdoensis* cuttings, combining leaf physiological measurements with transcriptome sequencing to examine their impact on growth development and gene expression. The results indicated that BF treatment significantly inhibited plant height, leaf length, and leaf width, while simultaneously increasing leaf palisade tissue thickness, upper epidermis thickness, and structural density. GF treatment exhibited opposite effects. Physiologically, GF treatment increased Gs and Ci, while BF treatment enhanced *WUE*. Both treatments increased Chlb and Car content, with BF treatment additionally raising total soluble sugars while reducing sucrose and starch content. Transcriptome analysis revealed significant DEGs related to sucrose and starch metabolism, plant hormone signal transduction, porphyrin and chlorophyll metabolism, and photoreceptors across the three treatments: BF treatment upregulated most genes in the sucrose and starch metabolic pathways, along with auxin pathway genes (*AUX/IAA*, *GH3*, *SAUR*); meanwhile, the GF treatment up-regulated most genes in the porphyrin and chlorophyll metabolism pathways and gibberellin pathway genes, while significantly inducing the expression of photoreceptor genes *HYH*, *COP1*, *CRY1*, *HY5*, and *PIF4*. Conversely, the BF treatment suppressed the expression of these genes. The findings of this study provide theoretical support for elucidating the evolutionary mechanisms by which plants adapt to different light environments.

## Data Availability

Transcriptome raw sequencing data files are available in the NCBI GEO database with project accession GSE269248.

## Supplementary Materials

The following supporting information can be downloaded at: https://www.mdpi.com/article/10.3390/biology14121658/s1, Table S1: The primers of seven differentially expressed genes used for qRT-PCR verification; Table S2: Summary of the RNA-Seq results; Table S3: The significantly enriched top 20 GO term and corresponding ID of the differentially expressed genes (DEGs); Table S4: List of significantly enrichment pathways by DEGs and the identifier of DEGs; Table S5: Annotation of DEGs in the Pathway.

## Abbreviations

The following abbreviations are used in this manuscript:

WF	Transparent colorless film
BF	Blue film
GF	Green film
Gs	Stomatal conductance
Ci	Intercellular CO_2_ concentration
WUE	Water use efficiency
Chla	Chlorophyll a
Chlb	Chlorophyll b
Car	Carotenoids
DEGs	Differentially expressed genes
FDR	False discovery rate
